# Differences in sensationalism in international news media reporting of COVID-19: An exploratory analysis using the Global Public Health Intelligence Network (GPHIN) system

**DOI:** 10.14745/ccdr.v51i23a05

**Published:** 2025-02-12

**Authors:** Joanna Przepiorkowski, Tenzin Norzin, Abdelhamid Zaghlool, Florence Tanguay, Dorcas Taylor, Victor Gallant, Linlu Zhao

**Affiliations:** 1 Global Public Health Intelligence Network, Public Health Agency of Canada, Ottawa ON

**Keywords:** event-based surveillance, COVID-19, sensationalism, public health emergency of international concern, public health intelligence, media

## Abstract

**Background:**

The Global Public Health Intelligence Network (GPHIN) is an event-based surveillance platform that collects thousands of pieces of open-source information, including international news media, across multiple languages on a daily basis. Analysts have observed that news media reporting in some languages tended to use more sensational wording to describe major health events. There has been minimal research exploring potential differences in sensationalism in international news media reporting to confirm these observations.

**Objective:**

This exploratory study assessed the differences in the level of sensationalism in early international news media reporting of COVID-19 through a mixed-methods analysis.

**Methods:**

Relevant news media articles received in GPHIN seven days following the Public Health Emergency of International Concern declaration of COVID-19 by the World Health Organization were extracted for screening and analysis. An adapted tool was used to measure the sensationalism of pandemic-related health news. Deductive thematic analysis was conducted to examine themes of sensationalism. Differences in prevalence of sensationalism in news media reporting by language and country/territory of publication were assessed. Sentiment analysis assessed the sentiment and emotional tone of the news media articles.

**Results:**

Of 951 news articles that met the eligibility criteria, 155 contained sensationalism. There were significant differences between languages (French, Russian and Spanish) and various domains of sensationalism. This study also found a more negative emotional tone in news media articles with sensationalism.

**Conclusion:**

This exploratory study showed that language has the potential to impact the perception of health events using more sensationalized language.

## Introduction

On January 7, 2020, Chinese authorities identified a novel coronavirus temporarily named “2019-nCoV” ((1)), which rapidly spread around the world. The World Health Organization (WHO) declared a public health emergency of international concern (PHEIC) on January 30, 2020. Due to the global spread of the virus, which came to be known as COVID-19, WHO characterized the outbreak as a pandemic on March 11, 2020 ((1)).

This pandemic was the first public health event of its kind with constant media coverage ((2)), becoming a political battleground, with leaders debating over public policy and medical interpretations ((2)). The pandemic also highlighted multiple social, cultural and economic issues arising from the media’s constant dissemination of information ((3)). Verified, official information, based on best information available at the time, was complicated by inaccurate claims amplified on various news media platforms, which proved to be almost as much of a threat to global public health as the virus itself ((4)).

Technological advancements and online news media create opportunities to keep people informed, connected and safe ((4)). However, it can also create the opportunity for sensationalizing issues by presenting news as more extraordinary, interesting or relevant than is objectively warranted, which can undermine global responses and jeopardize measures to control major public health events ((4)).

During global disasters such as pandemics, crisis communication is crucial to dispel fears and uncertainties and unify individuals worldwide against public health threats ((5)). Sensational communication, however, can result in negative personal and economic consequences ((5)). For new or emerging diseases, particularly when there is limited available information from official sources, sensational reporting may influence the risk assessment and response to the event implemented by decision-makers, as well as the perception of the risk of the event by the public ((6,7)).

The Global Public Health Intelligence Network (GPHIN) is an all-hazards event-based surveillance system that is operated by the Public Health Agency of Canada (PHAC) ((8)). The system was developed by the Government of Canada in collaboration with the WHO for use by non-governmental agencies and organizations, as well as government authorities who conduct public health surveillance ((9)). To identify potential public health threats, GPHIN collects and assesses thousands of pieces of open-source information on a daily basis through artificial intelligence algorithms (i.e., machine learning, natural language processing). Although GPHIN monitors a diverse array of open sources, most of the information is currently sourced from news media (i.e., news media in the context of this study refers to mass media that focus on delivering news in text format via the Internet to the public) ((8)). The information is then curated by a multicultural team of analysts, covering 10 languages (Arabic, Chinese [Simplified], Chinese [Traditional], English, Farsi, French, Hindi, Portuguese, Russian and Spanish).

Given their linguistic and cultural diversity, GPHIN analysts add a language and cultural perspective to the interpretation of international reporting that may otherwise be misinterpreted or misunderstood if only machine-translated English or only a Canadian cultural lens is used. Over time, GPHIN analysts have observed that news media from various languages tended to use more exaggerated or hyperbolical expressions/terms to describe new or emerging diseases.

There has been minimal research exploring potential differences in sensationalism in international news media reporting of major health events to confirm these observations and presents a knowledge/research gap. To address this gap, this exploratory study assessed the differences in the level of sensationalism in early international news media reporting of COVID-19 through a mixed-methods analysis.

## Methods

Relevant news media articles received in the GPHIN system in the first seven days following the declaration of the COVID-19 PHEIC on January 30, 2020, were identified and extracted (**Table 1**). The time restriction of the first seven days was chosen to observe the initial reaction of news media following the COVID-19 PHEIC declaration, so there is a shared baseline for the globally relevant health event. Articles in Arabic, Chinese (Simplified and Traditional), English, Farsi, French, Portuguese, Russian and Spanish were reviewed (the Hindi language was not yet implemented into the GPHIN system at the time of the study). Reports from non-news media sources, including official sources such as the WHO, European Centre for Disease Prevention and Control, and United States Centers for Disease Control and Prevention were excluded.

**Table 1:** Global Public Health Intelligence Network system query/search strategy

Date	GPHIN system query	Search results (conducted on September 14, 2022)
Date of PHEIC declaration by the WHO: January 30, 2020 Date range of relevant news media articles received in the GPHIN system: January 30–February 6, 2020	(Title/Text contains (exact match): coronavirus OR Title/Text contains (exact match): corona virus OR Title/Text contains (exact match): 2019-nCoV OR Title/Text contains (exact match): Wuhan pneumonia) AND (Title/Text contains (exact match): PHEIC OR Title/Text contains (exact match): public health emergency of international concern OR Title/Text contains all of the following (comma separated): international, emergency) AND Date received between 2020-01-30 and 2020-02-06^a^	951

Abbreviations: GPHIN, Global Public Health Intelligence Network; PHEIC, public health event of international concern; WHO, World Health Organization

^a^ The COVID-19 and SARS-CoV-2 nomenclature were officially introduced by WHO on February 11, 2022, and therefore not included in the query (including them in the query did not change the search results)

An adapted version a tool by Hoffman *et al*. ((10)) was piloted to measure the sensationalism of pandemic-related health news was used to assess five domains of sensationalism as described in **Table 2**. For this study, a binary “Yes/No” response was used for the tool, instead of the five-point Likert-like scale, where “Yes” represented the presence of sensational text and “No” represented the absence of sensational text. This modification was made to avoid the potential subjectivity of assessing the relative degree of sensationalism using the Likert-like scale (where the differences between “not too much,” “somewhat” and “fairly” sensationalizing are open to interpretation). An article was deemed to have overall sensationalism if at least one of the domains listed in Table 2 was selected as “Yes.”

**Table 2:** Five domains of sensationalism tool^a^

Domain	Question
Exposing	Does the article attempt to expose certain events?
Speculating	Does the article offer a guess or suggest what the future consequences of an issue are likely to be?
Generalizing	Does the article make generalizing statements that extrapolate a trend out of an incident or pass a judgement about a whole class of people?
Warning	Does the article generate anxiety about an issue or offer suggestions on how to avoid becoming a victim?
Extolling	Does the article exaggerate facts as extraordinary, project events as historic, praise individuals for heroic acts, etc.?

^a^ Adapted from reference ((10))

An inclusion/exclusion assessment was performed using the criteria in Table 1 by two reviewers, with any disagreements resolved by consensus. The following data was extracted for each article included for analysis: assessment against each of the five domains of sensationalism, overall assessment of sensationalism (Sensationalism=Yes if at least one domain selected and Sensationalism=No if no domains were selected), date of publication, country/territory of news media outlet and original language of publication. Data extraction was performed by one reviewer and validated by a second reviewer.

The title and body of each news media article included for analysis were independently appraised for sensationalism by two reviewers, with any disagreements resolved by consensus. For non-English articles, English analysts reviewed the machine-translated text in English, while GPHIN analysts with expertise in the language of the article performed a secondary review in the original language.

This study used a mixed-method approach for analysis. For the qualitative portion, thematic analysis, a flexible method that enables the identification of patterns of meaning (themes) across data sets by interrogating both semantic and latent meanings (i.e., content, ideas, assumptions) below the surface ((11)), was used. In this study, deductive thematic analysis was used as themes were identified within each domain. There were 155 news media articles identified as having overall sensationalism and top themes within each domain were recorded.

For the quantitative portion of the study, the analysis of articles with overall sensationalism (Sensationalism=Yes) was performed using Stata IC 15.1. Differences in the prevalence of sensationalism in news media reporting by language were assessed using the chi-square test and Fisher’s exact test, depending on whether assumptions were met. Four sentiment analysis methods, AFINN, Bing, Syuzhet and National Research Council packages, were performed to assess the sentiment and emotional tone of news media articles ((12)). These sentiment analyses were done using algorithms implemented in R programming language (see **Table 3**). Using the Welch two sample t-test function in R, the sentiments were then compared between news media articles with (“Yes”) and without (“No”) overall sensationalism to determine whether there were statistical differences in the sentiment and tone of describing and reporting on COVID-19. The text analyzed in this analysis was strictly in English or machine-translated English due to R package restrictions.

**Table 3:** Sentiment analysis methods

Method	Summary
AFINN	AFINN is a lexicon-based sentiment analysis method. It assigns pre-defined sentiment scores (positive or negative) to individual words in a document. The scores for each word are then aggregated to calculate an overall sentiment score for the document. AFINN typically provides a numeric score for each document, where a higher score indicates a more positive sentiment and a lower score indicates a more negative sentiment.
Bing	Bing is another lexicon-based sentiment analysis method. It assigns positive, negative or neutral labels to individual words. Like AFINN, it aggregates the sentiment labels to calculate an overall sentiment score for a document. It is often used for binary sentiment classification (positive or negative).
Syuzhet^a^	Syuzhet is a sentiment analysis package in R that relies on sentiment lexicons and dictionaries. It provides a more fine-grained approach, categorizing sentiment into emotions such as joy, anger, sadness and overall sentiment. This method allows for a more nuanced emotional tone analysis and can provide insights into the specific emotions expressed in a document.
National Research Council Canada (NRC)	The NRC *Sentiment and Emotion Lexicons* tool is a more advanced approach considering a broader range of emotions and sentiments. It categorizes sentiment into various dimensions: positive, negative, anger, anticipation, disgust, fear, joy, sadness, surprise and trust. This method provides a rich understanding of the emotional content in a document, making it suitable for more detailed sentiment analysis.

^a^ Reference ((12))

## Results

### Screening

**Figure 1:**
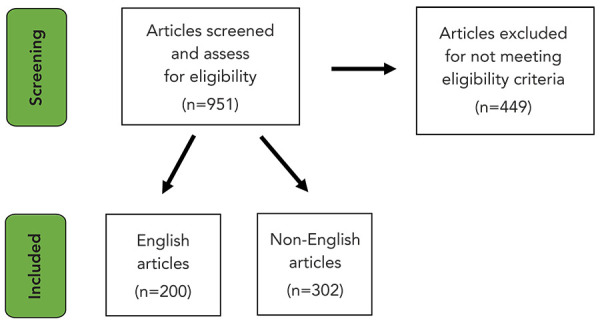
Screening of news media related to COVID-19 public health event of international concern on the Global Public Health Intelligence Network system

From the GPHIN system, 951 articles were screened and assessed for eligibility. Of these, 449 were excluded as they did not meet the eligibility criteria. There were 200 English and 302 non-English articles included in the analysis (**Figure 1**). Out of 502 articles, 155 were identified as having overall sensationalism. Sensationalism and news media country/territory of publication was not found to be statistically significant (**Appendix, ****Table A1**) and, therefore, we could not explore the potential differences between reporting in countries and assess whether it was a potential confounder.

### Qualitative analysis

Common themes observed within news media articles that had overall sensationalism are presented in **Table 4**.

**Table 4:** Themes identified within sensationalism domains in COVID-19 news media

Domain	Themes identified
Exposing	Criticism of China Negative feedback on how the situation is being handled Racism against the Chinese population Deficiencies in government system Sparse information being given from China Foreigners not being taken care of Local infrastructure being overwhelmed Mistrust over China’s handling of the situation China semi-quarantine “threat” to the global economy Situation could have been prevented Criticism of the United States Downplaying the virus’ severity Unnecessary or excessive restrictions on China Promoting panic through closing borders Inappropriately overreacted
Speculating	Unknown/grim outcome, unknown consequences Uncertainty looms Impact being difficult to assess Suspicions over the true cause of the virus and spread Future of virus being unpredictable COVID-19 may impact China’s economy/global economy (how will tourism/businesses be affected)
Generalizing	Discrimination of Chinese individuals and their “cause” of COVID-19 COVID being “less dangerous” than influenza/SARS Unnecessary/unjustified panic, not a major problem
Warning	Threat of COVID-19 spreading to other countries/worldwide Flu being more of a threat than COVID-19 (therefore, individuals should focus more on flu prevention) Threat of misinformation spreading Potential negative impact on China’s economy and moreover, to the global economy Whole world should be “on alert”
Extolling	Labelling COVID-19 as a monster/evil/demon/invisible killer Comparing the fight against COVID-19 to war Describing the situation as the end of the world: zombie apocalypse, ghostly city, devastating plague, doomsday predictions, pandemic deadlier than wars The notion of fear “spreading like a virus”

With each sensationalism domain, five statistically significant themes were observed.

**Exposing domain:** News media in the French language exposed that local healthcare systems were becoming overwhelmed and saturated with patients ((13)). There was also negative criticism of how China was handling the COVID-19 situation and how they tried to maintain an image to the global community, however the “social pressure was too much” ((14)).

**Speculating domain:** The French language news media had speculated about what would happen to the local business and economy if COVID-19 spread and shut down countries ((15)). Articles had also speculated about the true cause of COVID-19 and from where it came ((16)). They also questioned whether isolation measures ever worked ((17)). Similarly, news media in the Russian language speculated about whether COVID-19 would do harm to the economy ((18)).

**Generalizing domain:** Discriminatory undertones were found within new media in the French language. Articles stated that the cause of the situation was due to Chinese citizens and that it was Wuhan’s problem rather than a problem for the rest of the world ((14,19–21)).

**Warning domain:** For the articles with the elements of the warning domain, the focus was broad. Articles in the Spanish language warned about the situation of COVID-19 spreading to other countries and that COVID-19 was spreading much faster than the previous SARS outbreak in 2001 ((22–25)). Articles further warned readers that the situation in China was completely out of hand and that the issue of COVID-19 was extremely serious ((26)). There was also a notion that the virus was unstoppable, emphasizing the urgency and anxiety of the situation. A theme of warning, telling readers that they were dealing with a dangerous enemy, was also noted ((27–29)).

**Extolling domain:** The Russian language was the only language that was statistically significant by the extolling domain. Articles mentioned the “fight against evil” and that “a monster is born” for which “the world is not ready” ((30,31)).

### Quantitative analysis

**Figure 2:**
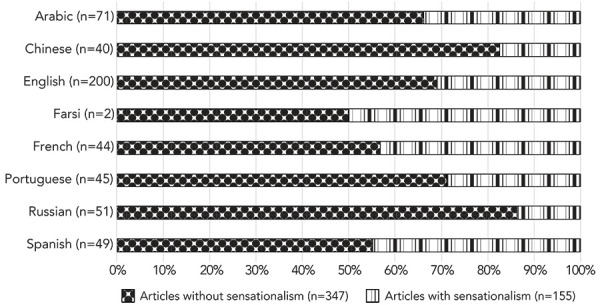
News media articles reviewed by language with and without overall sensationalism

**Sensationalism domains and language:** English and Arabic were identified as having the highest number of articles with overall sensationalism (n=62 and n=24, respectively), while Farsi, Russian and Chinese were found to have the lowest number of overall sensationalism (n=1, n=7 and n=7, respectively) (**Figure 2**). The French language was statistically significant for exposing (*p*=0.004), speculating (*p*=0.007) and generalizing (*p*=0.007). The Russian language was statistically significant for speculating (*p*=0.046), generalizing (*p*=0.046), warning (*p*=0.013), extolling (*p*=0.046) and overall sensationalism (*p*=0.004). The Spanish language was statistically significant for warning (*p*=0.034) and overall sensationalism (*p*=0.034).

**Sentiment analysis:** To determine whether differences in sentiment were statistically significant for the two groups (Overall Sensationalism=Yes vs. Overall Sensational=No), t-tests were performed using R programming.

· For the AFINN score comparison, t(240)=−3.8309, *p*<0.001

· For the Bing score comparison, t(235)=−4.7292, *p*<0.001

· For the Syuzhet score comparison, t(217)=−2.962, *p*<0.001

The *p*-value obtained from the tests suggested statistical significance in sentiment scores between the two groups. A more negative mean AFINN, Bing and Syuzhet score for the overall sensationalism news article group indicated a difference in the overall sentiment or emotional tone between these groups.

Regarding the National Research Council Canada’s (NRC) score for sentiments, the comparison was made to negative, positive and fear sentiments. The scores in the Overall Sensationalism=Yes news group were significantly higher in all three using the t-test to compare.

· The results for NRC-negative comparison: t(239)=5.483, df=239.67, *p*<0.001

· The results for NRC-positive comparison: t(247)=4.5944, *p*<0.001

· The results for NRC-fear comparison: t(254)=5.4729, *p*<0.001

The NRC sentiment scores for negative, positive and fear sentiments were significantly higher in the Overall Sensationalism=Yes news article group. This aligns with the expectation that sensationalism exaggerates emotions, including negative and fearful sentiments. The higher positive scores may be due to sensationalized content trying to elicit strong emotional reactions from readers.

Based on the provided results and analysis, there was a significant difference in sentiment and emotional tone between articles with sensationalism compared with those without sensationalism.

## Discussion

This study has demonstrated that even with machine translation, sensational language can still be understood, and this may have an influence on a reader’s perception of a given issue. Themes repeated in news media articles, regardless of language, may also impact and change the reader’s perceptions. Sensationalism in news media reporting could have impacted how COVID-19 was perceived after the PHEIC declaration. The analysis of sentiment scores indicated a clear and statistically significant difference in sentiment and emotional tone between sensational and non-sensational articles.

As seen in our study, media may use elements from warning, extolling, speculating and/or exaggerating domains of sensationalism to capture the reader’s attention and sway the reader in a particular direction. This could be damaging to a reader as, oftentimes, when reading a news article, they may not be able to do anything to prevent or reduce the issue’s risk, which could increase perceived vulnerability, creating anxiety ((32)). This idea complements further evidence suggesting that the more access one has to information, the more stressful one may become, potentially inducing unnecessary fear and concern ((33)).

Our study did not look at the effects of the use of sensationalism in social media; however, our findings on traditional news media complement research on both social media and traditional news media and the potential misperception of information regarding COVID-19. A study by Montezari *et al.* highlighted that exposure to COVID-19 news on social media was significantly correlated with increased feelings of anxiety and fear, as well as behavioural changes ((32)). Ravenelle *et al*. observed that increased media consumption was linked to decreased mental health and a sense of unhealthiness ((34)). Other research studies have found that even if one has a highly curated social media feed, there is still a possibility for media to contain misconstrued messages and information ((35,36)). Dechene *et al.* noted that with increasing cumulative exposure to exaggerated information, users are more likely to experience a “reinforcement effect,” where familiarity leads to a stronger change and belief of opinion ((37)).

## Limitations

A limitation of this exploratory study was that the articles included for review were restricted to those picked up through the GPHIN system and therefore may not be representative of all available news media articles available online. Misclassification bias could have occurred when reviewing articles for sensationalism. We note that the analysis performed was only looking at the seven days following the PHEIC; therefore, we were unable to conduct a trend analysis over time to see if there was a change in the language used in the reporting of COVID-19 by the media. Although efforts were made to minimize translation and cultural bias by including language specialists in the sensationalism assessments, there was likely residual language/cultural bias due to the study team living in Canada and working in English/French. The validity of the pilot tool used in this exploratory study to assess sensationalism has not yet been established.

## Conclusion/future research

Having ready access to international news media reporting allows individuals to be informed and connected; however, as demonstrated in this study, news media sources may be prone to sensationalism and should be interpreted with caution. The findings of this exploratory study suggest that it would be beneficial for tools to be developed that can help analysts and users of event-based surveillance systems flag potentially sensational articles so that the information could be appropriately assessed and used to inform decision-making. Such tools do not currently exist, although there are similar tools (e.g., websites such as mediabiasfactcheck.com) that provide information on political standpoints and the trustworthiness of news media sources. Additional research is needed to refine and validate tools for assessing sensationalism in news media and other media, as well as to examine the topic across different health events and time periods to identify broader trends.

## Appendix

**Table A1:** Proportion of articles by language and country/territory of publication (n=502)

Country/territory of publication	Languages							
	Arabic n (%)	Chinese n (%)	English n (%)	Farsi n (%)	French n (%)	Portuguese n (%)	Russian n (%)	Spanish n (%)
Argentina	-	-	-	-	-	-	-	3 (6.1)
Australia	-	-	10 (5.0)	-	3 (6.8)	-	-	-
Austria	-	-	-	-	-	1 (2.2)	-	-
Bahrain	1 (1.4)	-	-	-	-	-	-	-
Bangladesh	-	-	2 (1.0)	-	-	-	-	-
Brazil	-	-	1 (0.5)	-	-	20 (44.4)	-	-
Canada	-	-	13 (6.5)	-	-	-	-	-
Chile	-	-	-	-	-	-	-	3 (6.1)
China	5 (7.0)	4 (10.0)	2 (1.0)	-	1 (2.2)	-	2 (3.9)	-
Czechia	-	-	-	1 (50.0)	-	-	-	-
Egypt	11 (15.4)	-	-	-	-	-	-	-
France	7 (9.8)	2 (5.0)	-	-	30 (68.1)	-	-	2 (4.0)
Gaza Strip and West Bank	2 (2.8)	-	-	-	-	-	-	-
Germany	4 (5.6)	1 (2.5)	3 (1.5)	-	-	-	-	-
Hong Kong	-	15 (37.5)	-	-	-	-	-	-
India	-	-	11 (5.5)	-	-	-	-	-
Indonesia	-	-	1 (0.5)	-	-	-	-	-
Iran	-	-	-	1 (50.0)	-	-	-	-
Iraq	3 (4.2)	-	-	-	-	-	-	-
Ireland	-	-	3 (1.5)	-	-	-	-	-
Jordan	3 (4.2)	-	-	-	-	-	-	-
Kuwait	4 (5.6)	-	-	-	-	-	-	-
Latvia	-	-	-	-	-	-	1 (1.9)	-
Lebanon	1 (1.4)	-	-	-	-	-	-	-
Libya	1 (1.4)	-	-	-	-	-	-	-
Luxembourg	-	-	-	-	1 (2.2)	-	-	-
Malaysia	-	-	4 (2.0)	-	-	-	-	-
Mauritania	-	-	-	-	1 (2.2)	-	-	-
Mauritius	-	-	-	-	1 (2.2)	-	-	-
Mexico	-	-	-	-	-	1 (2.2)	-	3 (6.1)
Morocco	1 (1.4)	-	-	-	-	-	-	-
Netherlands	-	-	1 (0.5)	-	-	-	-	-
New Zealand	-	-	1 (0.5)	-	-	-	-	-
Oman	2 (2.8)	-	-	-	-	-	-	-
Pakistan	-	-	4 (2.0)	-	-	-	-	-
Peru	-	-	-	-	-	-	-	4 (8.1)
Philippines	-	-	7 (3.5)	-	-	-	-	-
Portugal	-	-	-	-	-	9 (20.0)	-	-
Qatar	7 (9.8)	-	10 (5.0)	-	-	-	-	-
Republic of Congo	-	-	3 (1.5)	-	-	-	-	-
Russia	-	-	2 (1.0)	-	-	-	37 (72.5)	4 (8.1)
Saudi Arabia	6 (8.4)	-	-	-	-	-	-	-
Singapore	-	2 (5.0)	4 (2.0)	-	-	-	-	-
South Korea	-	5 (12.5)	2 (1.0)	-	-	-	-	-
Spain	-	-	1 (0.5)	-	-	1 (2.2)	-	17 (34.6)
Switzerland	-	-	-	-	2 (4.5)	-	-	-
Syria	1 (1.4)	-	-	-	-	-	-	-
Taiwan	-	7 (17.5)	1 (0.5)	-	-	-	-	-
Tunisia	1 (1.4)	-	-	-	2 (4.5)	-	-	-
Turkey	-	-	1 (0.5)	-	1 (2.2)	-	-	-
Ukraine	-	-	-	-	-	-	11 (21.5)	-
United Arab Emirates	4 (5.6)	-	-	-	-	-	-	-
United Kingdom	5 (7.0)	-	40 (20.0)	-	-	-	-	2 (4.0)
United States	2 (2.8)	-	67 (33.5)	-	-	13 (28.8)	-	11 (22.4)
Unknown	-	4 (10.0)	4 (2.0)	-	2 (4.5)	-	-	-
Vietnam	-	-	2 (1.0)	-	-	-	-	-
Total	71	40	200	2	44	45	51	49

Abbreviation: -, not applicable

## References

[1] World Health Organization. Coronavirus disease (COVID-19). Geneva, CH: WHO. https://www.who.int/europe/health-topics/coronavirus

[2] Nelson T, Kagan N, Critchlow C, Hillard A, Hsu A. The Danger of Misinformation in the COVID-19 Crisis. Mo Med 2020;117(6):510–2. 33311767 PMC7721433

[3] Anwar A, Malik M, Raees V, Anwar A. Role of Mass Media and Public Health Communications in the COVID-19 Pandemic. Cureus 2020;12(9):e10453. 10.7759/cureus.10453 33072461 PMC7557800

[4] Volkmer I. Social media and COVID-19: A global study of digital crisis interaction among Gen Z and millennials. Melbourne, AU: University of Melbourne; 2021. 10.46580/124367

[5] Su Z, McDonnell D, Wen J, Kozak M, Abbas J, Šegalo S, Li X, Ahmad J, Cheshmehzangi A, Cai Y, Yang L, Xiang YT. Mental health consequences of COVID-19 media coverage: the need for effective crisis communication practices. Global Health 2021 Jan;17(1):4. 10.1186/s12992-020-00654-4 33402169 PMC7784222

[6] Pratama AR, Firmansyah FM. COVID-19 mass media coverage in English and public reactions: a West-East comparison *via* Facebook posts. PeerJ Comput Sci 2022;8:e1111. 10.7717/peerj-cs.1111 36262131 PMC9575862

[7] Ottwell R, Puckett M, Rogers T, Nicks S, Vassar M. Sensational media reporting is common when describing COVID-19 therapies, detection methods, and vaccines. J Investig Med 2021;69(6):1256–7. 10.1136/jim-2020-001760 34021053

[8] Public Health Agency of Canada. About GPHIN. Ottawa, ON: PHAC. https://gphin.canada.ca/cepr/aboutgphin-rmispenbref.jsp

[9] Rees EE, Ng V, Gachon P, Mawudeku A, McKenney D, Pedlar J, Yemshanov D, Parmely J, Knox J. Risk assessment strategies for early detection and prediction of infectious disease outbreaks associated with climate change. Can Commun Dis Rep 2019;45(5):119–26. 10.14745/ccdr.v45i05a02 31285702 PMC6587687

[10] Hoffman SJ, Justicz V. Automatically quantifying the scientific quality and sensationalism of news records mentioning pandemics: validating a maximum entropy machine-learning model. J Clin Epidemiol 2016;75:47–55. 10.1016/j.jclinepi.2015.12.010 26854419 PMC7127105

[11] Braun V, Clarke V. Thematic analysis. In Cooper H, Camic PM, Long DL, Panter AT, Rindskopf D, Sher KJ, editors. APA handbook of research methods in psychology, Vol. 2: Research designs: Quantitative, Qualitative, Neuropsychological, and Biological. Washington, DC: American Psychological Association; 2012. p. 57–71.

[12] Jockers M. Package ‘syuzhet’. Extracts Sentiment and Sentiment-Derived Plot Arcs from Text 2023. https://cran.r-project.org/web/packages/syuzhet/syuzhet.pdf

[13] Coronavirus: plus de 300 décès depuis le début de l'épidémie dont le premier hors de Chine. Liberation.fr; 2020. https://gphin.canada.ca/cepr/showarticle.jsp?docId=1006227894

[14] Dagorn D, Baruch J, Maad A, Benkimoun P, Lemaître F, Damage M, Hoorman C. Coronavirus epidemic: answers to 40 questions from readers of ”Le Monde”. Le Monde; 2020. https://www.lemonde.fr/les-decodeurs/article/2020/02/01/epidemie-de-2019-ncov-les-reponses-aux-40-questions-des-lecteurs-du-monde_6028082_4355770.html

[15] Coronavirus: plus de 300 décès depuis le début de l’épidémie dont le premier hors de Chine. Liberation.fr; 2020. https://gphin.canada.ca/cepr/showarticle.jsp?docId=1006233012

[16] Guitton-Boussion J. Coronavirus, an epidemic fueled by airplanes and climate change. Reporterre; 2020. https://reporterre.net/Le-coronavirus-une-epidemie-favorisee-par-l-avion-et-le-dereglement-climatique

[17] Morozov C. Coronavirus psychosis: “panic fear is the most dangerous thing”. Sputnik Afrique; 2020. https://fr.sputniknews.africa/20200131/psychose-autour-du-coronavirus-la-peur-panique-cest-ce-quil-y-a-de-plus-dangereux-1042983107.html

[18] Коронавирус опасен для экономики тем, что Китай имеет большое влияние на мировую промышленность. Mirror Weekly; 2020. [In Russian only]. https://gphin.canada.ca/cepr/showarticle.jsp?docId=1006223553

[19] Miserez M. Is the world right to be afraid of the Wuhan coronavirus? Swissinfo.ch; 2020. https://www.swissinfo.ch/fre/2019-ncov_le-monde-a-t-il-raison-d-avoir-peur-du-coronavirus-de-wuhan-/45531082

[20] Denmamode Y, Carpayen S. Coronavirus: Racisme under the microscope. Lexpress.mu; 2020. https://lexpress.mu/article/369314/coronavirus-racisme-sous-microscope

[21] Bondaz A. Coronavirus: How Beijing is trying to save face. The Conversation; 2020. https://theconversation.com/coronavirus-comment-pekin-cherche-a-sauver-la-face-131098

[22] The new coronavirus is now more lethal than the SARS outbreak in China. RT; 2020. https://actualidad.rt.com/actualidad/341861-nuevo-coronavirus-letal-brote-sars

[23] Telemundo. Coronavirus spreads to more countries, while another disease infects thousands of Americans. El Diario Ny; 2020. https://eldiariony.com/2020/01/31/el-coronavirus-alcanza-mas-paises-mientras-otra-enfermedad-contagia-a-miles-de-estadounidenses/

[24] Corbella J. La OMS se opone a las restricciones de viajes por el coronavirus. La Vanguardia; 2020. [In Spanish only]. https://www.lavanguardia.com/vida/20200131/473223306200/oms-opone-restricciones-viajes-coronavirus.html

[25] US and Japan advise against travelling to China after WHO global alert. Cinco Dias; 2020. https://cincodias.elpais.com/cincodias/2020/01/31/economia/1580455715_199678.html

[26] DerBlauEmond. Everything we haven’t been told about the Coronavirus, which could wipe out 1% of China’s economic growth. El Blog Salmon; 2020. https://www.elblogsalmon.com/entorno/todo-que-no-nos-han-contado-coronavirus-que-puede-hacer-vaporizarse-1-crecimiento-economico-china

[27] Coronavirus: Death toll from Wuhan virus rises to 350. La Republica; 2020. https://larepublica.pe/mundo/2020/01/31/coronavirus-en-vivo-minuto-a-minuto-del-virus-de-china-sintomas-cuantos-muertos-hay-mapa-en-tiempo-real-y-en-que-paises-esta-la-enfermedad-en-el-mundo-ultimas-noticias

[28] In record time: Chinese experts developed a rapid detection kit for coronavirus. Cooperativa.cl; 2020. https://www.cooperativa.cl/noticias/sociedad/salud/en-tiempo-record-expertos-chinos-desarrollaron-un-kit-de-deteccion/2020-01-31/054357.html

[29] Mamedyarov Z, Obukhova E, Labykin A. Дракон против демона. Expert; 2020. [In Russian only]. https://gphin.canada.ca/cepr/showarticle.jsp?docId=1006233248

[30] Чехия готова помочь КНР в борьбе с коронавирусом - президент. Uniform News; 2020. [In Russian only]. https://gphin.canada.ca/cepr/showarticle.jsp?docId=1006231115

[31] «Раздутая паника»: о признании коронавируса международной ЧС. Regnum.ru; 2020. [In Russian only]. https://gphin.canada.ca/cepr/showarticle.jsp?docId=1006223553

[32] Montazeri A, Mohammadi S, M Hesari P, Yarmohammadi H, Bahabadi MR, Naghizadeh Moghari F, Maftoon F, Tavousi M, Riazi H. Exposure to the COVID-19 news on social media and consequent psychological distress and potential behavioral change. Sci Rep 2023;13(1):15224. 10.1038/s41598-023-42459-6 37710006 PMC10502066

[33] Lee Y, Jeon YJ, Kang S, Shin JI, Jung YC, Jung SJ. Social media use and mental health during the COVID-19 pandemic in young adults: a meta-analysis of 14 cross-sectional studies. BMC Public Health 2022;22(1):995. 10.1186/s12889-022-13409-0 35581597 PMC9112239

[34] Ravenelle AJ, Newell A, Kowalski KC. “The Looming, Crazy Stalker Coronavirus”: Fear Mongering, Fake News, and the Diffusion of Distrust. Socius 2021;7:1–13. 10.1177/23780231211024776

[35] Feezell JT. Agenda Setting through Social Media: The Importance of Incidental News Exposure and Social Filtering in the Digital Era. Polit Res Q 2018;71(2):482–94. 10.1177/1065912917744895

[36] Fletcher R, Nielsen RK. Are people incidentally exposed to news on social media? A comparative analysis. New Media Soc 2018;20(7):2450–68. 10.1177/1461444817724170

[37] Dechêne A, Stahl C, Hansen J, Wänke M. The truth about the truth: a meta-analytic review of the truth effect. Pers Soc Psychol Rev 2010;14(2):238–57. 10.1177/1088868309352251 20023210

